# Interfacial Hydrolysis of Acetals on Protonated TEMPO-oxidized Cellulose Nanofibers

**DOI:** 10.1038/s41598-018-23381-8

**Published:** 2018-03-22

**Authors:** Yuya Tamura, Kyohei Kanomata, Takuya Kitaoka

**Affiliations:** Department of Agro-Environmental Sciences, Graduate School of Bioresource and Bioenvironmental Sciences, Kyushu University, 6-10-1 Hakozaki, Higashi-ku, Fukuoka, 812-8581 Japan

## Abstract

2,2,6,6-Tetramethylpiperidine-1-oxyl (TEMPO)-oxidized cellulose nanofibers (TOCNs), which have a high-density of exposed carboxylic acid groups on their crystalline surfaces, effectively act as acid catalysts in acetal hydrolysis. Carboxy-free cellulose nanofibers, polymeric carboxylic acids, and homogeneous acetic acid do not show significant catalytic activity under the same reaction conditions. Mercerized TOCNs differing from the original TOCNs in a crystalline structure were also ineffective, which suggests that the unique nanoarchitectural features of TOCNs, such as regularly aligned carboxylic acid groups, large specific surface areas, and structural rigidity, must be major factors in the acceleration of acetal hydrolysis. Kinetic analysis suggested that substrates and/or acid catalyst species were concentrated on the TOCN crystalline surfaces, which significantly enhanced the catalytic activity.

## Introduction

Cellulose nanofibers (CNFs) are promising natural nanomaterials for achieving a sustainable society because of their abundance, renewability, and attractive physicochemical properties for advanced materials applications^1–6^. Native cellulose consists only of β-1,4-linked anhydro-d-glucose repeating units, and assembles to form highly crystalline cellulose microfibrils of width ca. 4 nm, which form bundles of micrometer-scale fibers in wood cell walls^7,8^. *In vitro* nanofibrillation of these *in vivo*-formed bundled fibers affords CNFs. Various methods for CNF production have been reported, including mechanical, enzymatic, and chemical treatments^9^. CNFs have excellent physical properties such as high mechanical strength, transparency, and thermal dimensional stability; they are used in various high-performance materials such as gas barrier films^10–13^, composite fillers^14,15^, aerogels^16–19^, and electronic devices^20,21^.

Heterogeneous catalysis has inherent advantages in green and sustainable chemistry because of their ease of handling in practical applications^22–24^. However, they are generally less active than the corresponding homogeneous catalysts because of poor accessibility of substrates to active sites and negative impacts on the chemical microenvironment around the active centers. CNFs are attractive supporting materials for heterogeneous catalysts, especially for metal nanoparticles, because of their designable functions, large surface areas, and high chemical resistances^25^. Furthermore, the structural rigidity of CNFs significantly prevents aggregation of nano-sized catalysts, resulting in excellent catalytic efficiency in a wide range of reactions^26–29^. Among reported CNFs, 2,2,6,6-tetramethylpiperidine-1-oxyl (TEMPO)-oxidized cellulose nanofibers (TOCNs) are one of the most interesting supporting materials. TEMPO-mediated oxidation selectively converts primary alcohols to carboxylates at the C6-position of glucopyranose units on the surfaces of native cellulose I microfibrils^30^. The resultant carboxylates are exposed on the outer faces of crystalline cellulose I, providing ideal scaffolds for supporting metal nanoparticles. The use of highly dispersed TOCNs on which bare metal nanoparticles as catalysts in a number of reactions were supported has been reported^31–35^.

Although a number of catalytic reactions have been performed with metal nanoparticles immobilized on CNFs, few attempts have been made to use CNFs themselves as catalysts, despite their designable functionalities. Serizawa and co-workers used the abundant hydroxy groups on the surfaces of whisker-shaped CNFs to catalyze hydrolysis reactions of esters, monophosphates, and peptides. However, several days were required for the reaction to proceed to a significant level^36,37^. Chitosan and surface-deacetylated chitin nanofibers, which are amino analogs of CNFs, are also useful platforms for functional moieties. Aerogels of these nanofibers successfully catalyze Knoevenagel condensation^38^ and aldol reactions^39,40^. TOCNs have never been explored as Brønsted acid catalysts, to the best of our knowledge, although they have high-density carboxy groups on their crystalline surfaces. The development of TOCNs as heterogeneous acid catalysts is a promising approach, particularly in view of the increasing importance of solid acid catalysis^41–44^, because cellulose potentially provides green and sustainable catalysts derived from biomacromolecular resources such as wood.

**Figure 1:**
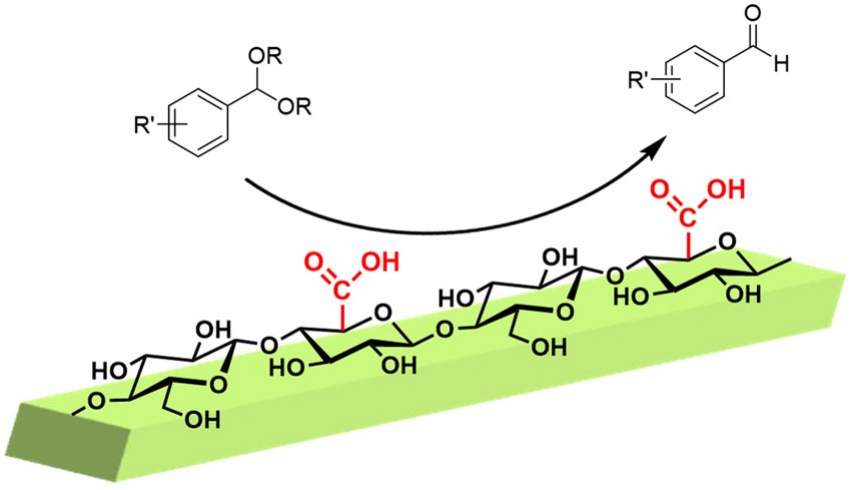
Schematic diagram of hydrolysis of dimethoxymethylbenzene on TOCN crystalline surface.

In the present study, protonated TOCNs were prepared and their use as Brønsted acid catalysts (Fig. 1) in the acid hydrolysis of various acetals was investigated. Acetal hydrolysis was conducted in semi-aqueous media, and the catalytic activity of the TOCNs was better than those of homogeneous or other conventional polymeric carboxylic acids. The kinetic profiles of the reaction and the effect of the carboxylate density of the TOCNs on the catalytic performance are discussed in terms of enhancement of the catalytic efficiency in the interfacial hydrolysis reactions of acetals.

## Results and Discussion

### Acid hydrolysis of dimethoxymethylbenzene by TEMPO-oxidized cellulose nanofibers

**Figure 2:**
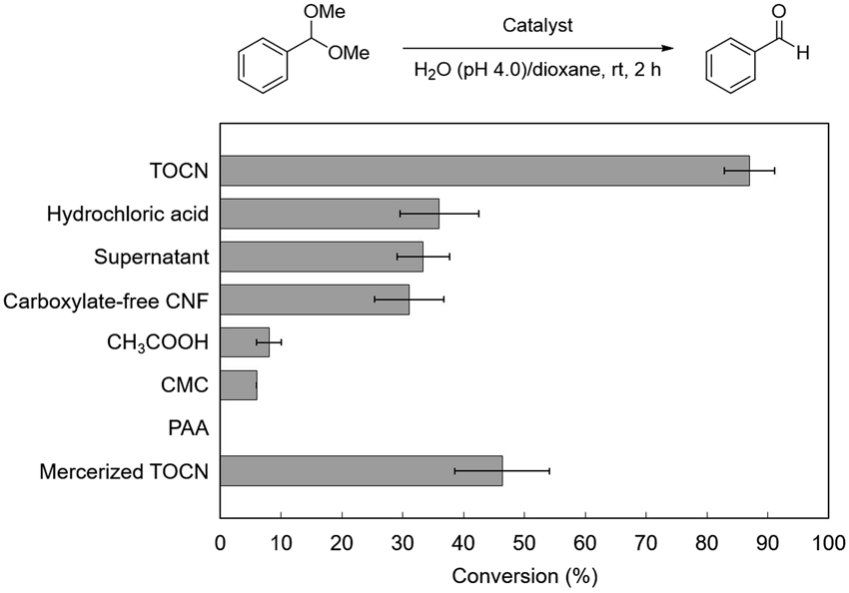
Hydrolysis of dimethoxymethylbenzene using various acid catalysts.

The acid hydrolysis reactions of dimethoxymethylbenzene were performed in a 1:1 (v/v) mixture of water adjusted to pH 4.0 and dioxane at room temperature. Benzaldehyde was the sole product in all cases (Fig. 2). The reaction proceeded rapidly in the presence of protonated TOCNs (COOH = 1.57 mmol g^−1^ of TOCNs, 17 mol% of carboxylic acid with respect to dimethoxymethylbenzene), and the substrate was consumed almost completely within 2 h. In contrast, neither an HCl solution of pH 4.0 nor the supernatant of pH 4.0 obtained by complete removal of TOCNs from the mixture was effective in the hydrolysis reaction, clearly suggesting the unique features of TOCNs as a heterogeneous acid catalyst. The difference in conversion was far significant at pH 4.4, where the effect of the residual HCl is almost negligible in this case (Figure S2a). Furthermore, physically pulverized CNFs with no carboxylic acid groups also showed low catalytic efficiency. These results presumably indicate that the dense interfacial carboxy groups on the TOCN crystalline surfaces play a crucial role in accelerating the hydrolysis reaction. The performance of the TOCN catalyst was superior to that of homogeneous acetic acid at the same pH. The comparison between TOCNs and acetic acid with the same molar catalyst loading also revealed higher catalytic efficiency of TOCNs, where the consequent pH values were 4.0 for TOCNs and 3.2 for acetic acid (Figure S2b). Therefore, the catalytic efficiency of the TOCNs is regarded as being much higher than that of homogeneous acetic acid. Some polymeric carboxylic acids were also examined for comparison. Carboxymethylcellulose (CMC) and poly(acrylic acid) (PAA) were used as typical water-soluble polymers with flexible molecular structures having carboxylic acids; neither of them was effective in this hydrolysis reaction. The low catalytic efficiencies of CMC and PAA can be attributed to aggregation of active acid sites susceptible to hydrophobic solvents such as dioxane. In contrast, TOCNs are crystalline nanofibers consisting of bundled cellulose chains. The rigid and high-aspect-ratio structures of TOCNs, often forming stable network structures in solvents, prevent undesirable aggregation^32^, resulting in effective contact of the surface carboxy groups with the reactants to enable such high catalytic performance. Furthermore, mercerized TOCNs, which have a typical cellulose II crystalline structure (COOH = 1.12 mmol g^−1^ of TOCNs; see Figure S3a for detailed characterization), were less effective in the present hydrolysis than the original TOCNs, which have the cellulose I crystalline structure. This result shows that the crystalline structure and/or crystallinity of the TOCNs would have a significant impact on the catalytic performance. We assume that the dense acidic centers on the rigid cellulose I crystalline surfaces possibly make some positive contribution to such unique catalytic enhancement, although it cannot be completely denied that the other carboxylic acids in the amorphous region contributed to the catalysis. It is worth noting that the TOCN crystallinity remained almost unchanged after the hydrolysis reaction; the β-1,4-glycosidic linkages in TOCN, which are also acetal functionalities, were not affected under the acidic reaction conditions used in this study (Figure S4).

### Kinetic study of TOCN-catalyzed acetal hydrolysis

**Figure 3:**
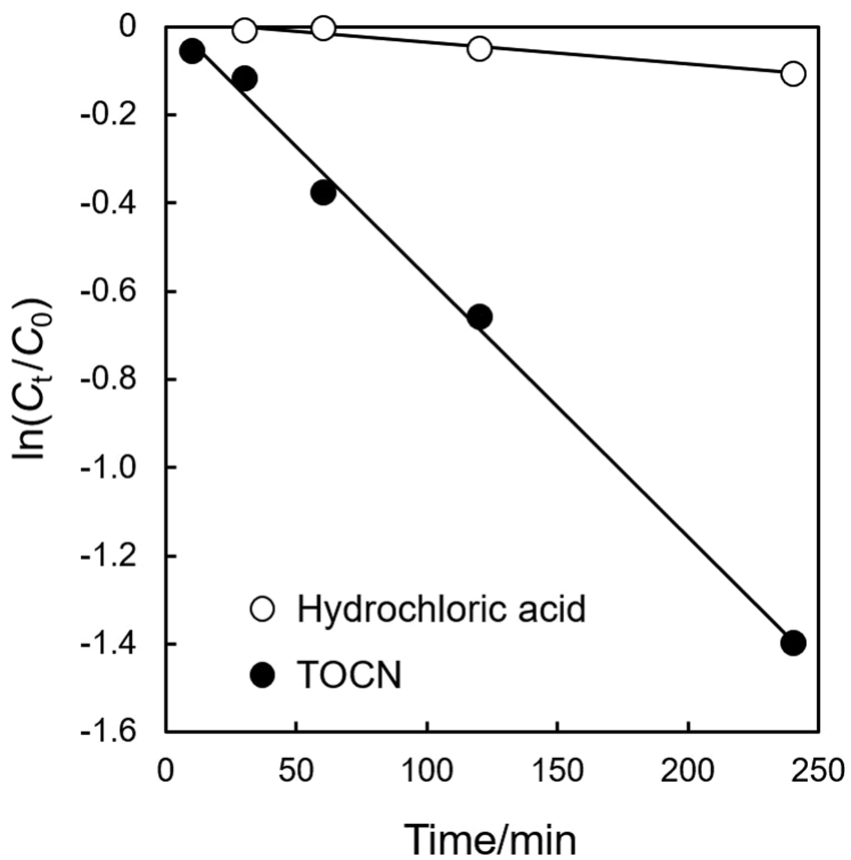
Plot of ln(*C*_*t*_/*C*_0_) versus reaction time for dimethoxymethylbenzene hydrolysis at pH 4.4 and 20 °C.

Further information on the catalytic behavior of TOCNs in the present hydrolysis reaction was obtained by examining the time course of the reaction at various pH values and temperatures (Table S2 and Figure S5). The results show a linear correlation between ln (*C*_*t*_/*C*_0_) and the reaction time, where *C*_0_ and *C*_*t*_ are the concentrations of dimethoxymethylbenzene at the initial and designated times, respectively. These results clearly indicate that dimethoxymethylbenzene hydrolysis follows pseudo-first-order kinetics. As shown in Fig. 3, the rate constants for the reaction with the TOCNs, which were calculated from the slopes, are up to 12 times higher than that with HCl at pH 4.4 (5.9 × 10^−3^ and 5.0 × 10^−4^ min^−1^ respectively). However, despite the large increase in the apparent rate constants in the presence of TOCNs, the activation energies of the reactions with TOCNs and HCl were similar (88.8 and 84.1 kJ mol^−1^, respectively; Figure S6 and Table S3). The rate constants derived from the activation energies are therefore also comparable, regardless of the presence of TOCNs. The reaction rate *v* is expressed as *v* = *k*[H^+^][*C*_*t*_]; hence the increase in the reaction rate caused by the TOCNs is attributed to an increase in the apparent concentrations of the reactants and/or acid catalyst species. The increase in the apparent concentrations indicates that the reactants are adsorbed to get together on the TOCN surfaces, where they are in direct contact with the carboxylic acid catalyst. In this regard, attractive interactions between cellulose and small aromatic compounds, including dimethoxymethylbenzene, were investigated in detail by using a combination of gas chromatographic analysis and molecular modeling^45,46^. Furthermore, aromatic amino acids such as tyrosine and tryptophan play an essential role in binding various glycohydrolases to cellulose surfaces^47–49^. In this study, such possible interactions could increase [*C*], and the accumulated carboxylic acids on the TOCNs could increase [H^+^] at the interface, eventually contributing to acetal hydrolysis. Although the precise mechanism at a molecular level is still unclear, kinetic analysis and the previously reported results described above suggest that attractive interactions between the reactants and the crystalline TOCN acid sites must be crucial in the present catalytic system.

### Effects of carboxy contents of TOCNs on the catalytic efficiency

Our results show that carboxylic acids on TOCNs provide an effective acid catalyst, therefore the effects of the amount and density of carboxy groups on the catalytic performance were investigated. A series of TOCNs with different carboxy contents were prepared from physically pulverized CNFs by TEMPO-mediated oxidation. TOCNs with carboxy contents ranging from 0.39 to 1.81 mmol g^−1^ of TOCNs were successfully prepared (Table S1). Transmission electron microscopy confirmed complete individualization of the cellulose microfibrils (Figure S7). The hydrolysis reactions of dimethoxymethylbenzene (Table 1) were carried out using the prepared TOCNs; the TOCN dosage was fixed, i.e., the molar amount of carboxy groups was different in each case. The reaction gave moderate conversion in the presence of TOCNs with a high carboxy content (Table 1, entry 1). When the carboxy content was decreased to 1.21 mmol g^−1^ of TOCNs, the catalytic efficiency clearly increased, leading to a higher conversion, although the acid loading in the reaction system was lower (Table 1, entries 1–3). This is probably because some hydrophobic interactions between dimethoxymethylbenzene and the TOCNs became relatively predominant as the amount of hydrophilic carboxy groups on the TOCN surfaces decreased. As mentioned before, attractive interactions of aromatic compounds with crystalline cellulose surfaces have been extensively studied^45–49^. Besides, it has been widely reported that the adsorption of the substrates to the catalyst surfaces is a critical factor in the catalytic performance in hydrolysis reactions catalyzed by solid acids such as zeolites and sulfonated carbon catalysts^50–53^. In other case, it was reported that amphiphilicity of TOCNs allowed the stable formation of oil-in-water Pickering emulsion^54,55^, implying the functionality of TOCNs for hydrophobic interactions in mixed solvents. Therefore, such possible interactions would be significant in interfacial catalysis, including the TOCN-mediated hydrolysis in this study. Further decreases in the carboxy content diminished the catalytic efficiency because the number of catalytically active acid sites was critically decreased (Table 1, entries 4 and 5). These results indicate that there is an optimal carboxy content, being closely related to the balance between catalyst loading on TOCN surfaces and their affinity with substrate molecules, which is suitable for acceleration of the reaction. The conversion remained high even at lower carboxy contents when the acid loading was kept constant (Table 1, entries 6–8). These results also confirm the importance of the TOCN surfaces and the amount of active catalytic sites on the TOCNs.

**Table 1:** Effect of TOCN carboxy content on catalytic efficiency.

Entry	Carboxy content/mmol g^−1^ of TOCNs	TOCN dosage/mg	Amount of COOH/mmol	Conversion/%
1	1.81	110	0.19	76
2	1.55	110	0.17	86
3	1.21	110	0.13	98
4	0.94	110	0.10	95
5	0.39	110	0.04	93
6	1.21	140	0.17	99
7	0.94	181	0.17	99
8	0.39	436	0.17	99

### Substrate scope of TOCN-catalyzed hydrolysis of acetals

**Figure 4:**
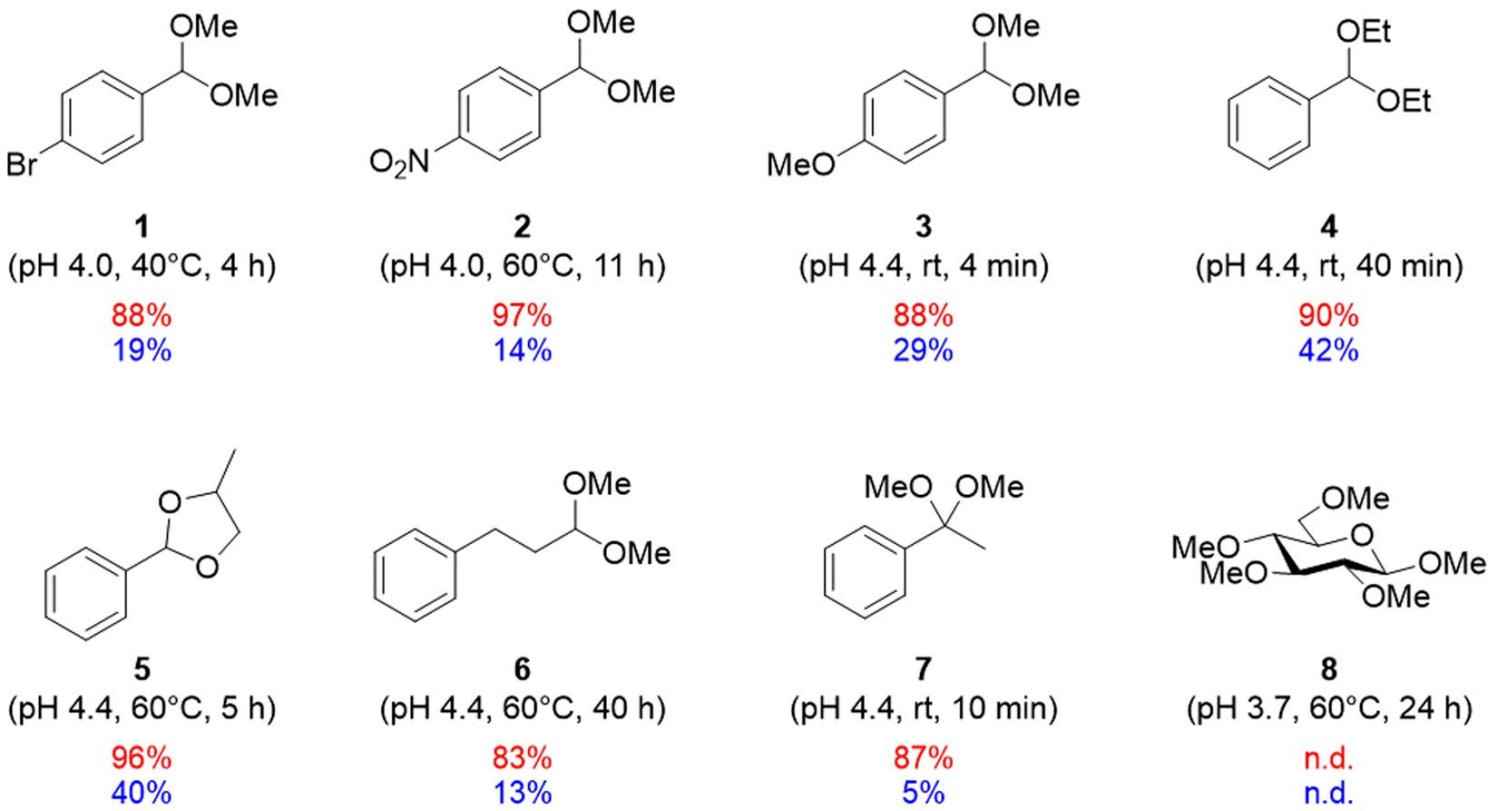
Substrate scope of acetal hydrolysis catalyzed by TOCNs (red) and HCl (blue). n.d.: not detected.

Once we had established that protonated TOCNs are a promising acid catalyst, we explored the substrate scope of the present TOCN-catalyzed hydrolysis reaction (Fig. 4). The TOCNs showed good catalytic efficiency in reactions with substrates bearing electron-withdrawing and electron-donating groups at the *para* position (**1**, **2**, and **3**). Diethylacetal **4**, cyclic acetal **5**, aliphatic acetal **6**, and ketal **7** were also smoothly hydrolyzed under mild reaction conditions in the presence of TOCNs, showing clear contrast against the TOCNs-free systems. Whereas, the hydrolysis reaction little proceeded for glucopyranoside **8**, being in agreement with the fact that the TOCN crystallinity remained almost unchanged after the hydrolysis reaction, although the TOCNs have the β-1,4-glycosidic linkages (Figure S4). These results again suggest that TOCNs are an outstanding catalyst in acid hydrolysis of various aromatic acetals.

## Conclusion

In summary, we showed that surface-carboxylated CNFs, i.e., TOCNs, greatly enhance acetal hydrolysis. The catalytic efficiency of the heterogeneous TOCN catalyst was far superior to those of other polymeric carboxylic acids or homogeneous acetic acid. Mercerized TOCNs, which have the cellulose II crystalline structure and amorphous zone, were less effective than the original TOCNs, which have the cellulose I structure with high crystallinity. This enhanced catalytic activity is attributed to the physicochemical properties and nanoarchitecture of the TOCNs: high-density catalytic sites, a large specific surface area, and structural rigidity, which prevents aggregation. The interfacial concentrations of reactants and/or acid catalyst sites would be significant in the catalytic mechanism. The present findings will open up a new phase in the investigation of novel functions of CNFs in green sustainable chemistry.

## Materials and Methods

### Materials

Softwood-derived TOCNs (1.1 wt%, COONa, 1.57 mmol g^−1^ of TOCNs) were supplied by Nippon Paper Industries Co., Ltd. (Tokyo, Japan). Carboxylate-free CNFs that had been physically pulverized with a high-speed water-jet machine (BiNFi-s, WMa-10002, 2.1 wt%) were purchased from Sugino Machine Limited (Uozu, Japan). The TOCNs supplied by Nippon Paper Industries Co., Ltd. were used in the acetal hydrolysis reactions shown in Figs 2, 3 and 4; the reactions used to obtain the data in Table 1 were performed using TOCNs with various carboxy contents, which were prepared in our laboratory by TEMPO-mediated oxidation of physically pulverized CNFs (BiNFi-s). Dimethoxymethylbenzene was purchased from Wako Pure Chemical Industries, Ltd. (Osaka, Japan) and used without further purification. Other chemicals and organic solvents were purchased from Sigma–Aldrich Japan (Tokyo, Japan), Wako Pure Chemical Industries, Ltd. (Osaka, Japan), and the Tokyo Chemical Industry Co., Ltd. (Tokyo, Japan), and used as received. The water used in this study was purified with an Arium Ultrapure Water System (Sartorius Co., Ltd., Tokyo, Japan). Full list of materials and chemicals with their grades and purity is shown in the Supplementary Information.

### Preparation of TOCNs

A series of TOCNs with different carboxy contents were prepared from physically pulverized CNFs (BiNFi-s WMa-10002, 2.1 wt% of water suspension, Table S1 and Figure S1). In brief, CNFs (6.0 g by dry weight) were suspended in water (300 mL) containing TEMPO (96 mg) and sodium bromide (600 mg). TEMPO-mediated oxidation was started by adding 2.0 M aqueous sodium hypochlorite solution to the suspension. The pH of the suspension was maintained at 10 by step-wise addition of 0.5 M aqueous NaOH solution, using an automatic pH titrator (Mitsubishi Chemical Analytech, Yamato, Japan). The oxidation was quenched by adding ethanol (6 mL) when the pH became constant. The oxidized CNFs were thoroughly washed with 0.1 M aqueous HCl solution and then water, by repeated centrifugation at 4170 *g* for 10 min (three times). The TOCNs were re-suspended in 500 mL of water and neutralized with 0.5 M aqueous NaOH solution until the pH was greater than 10. The suspension was subjected to the aqueous counter collision method^56^ at 245 MPa, using a high-pressure water-jet system equipped with a dual-nozzle chamber with nozzles of diameter 0.1 mm (Star Burst Labo, Sugino Machines Limited, Uozu, Japan). The concentration of the TOCN suspensions ranged from 0.4 wt% to 0.7 wt%. The carboxylate contents of the TOCNs were determined by electrical conductivity titration^57^. The resultant transparent dispersions of TOCNs were used in the acetal hydrolysis reactions after protonation.

### Hydrolysis reactions of acetals catalyzed by protonated TOCNs

A water suspension of TOCNs in sodium carboxylate form (1.1 wt%, 110 mg by dry weight) was mixed with 0.1 M aqueous HCl solution (20 mL); the mixture was shaken several times, followed by centrifugation at 4,300 *g* for 10 min. The supernatant was removed, the TOCN precipitate was washed with water, and the mixture was centrifuged again. This treatment was repeated until the supernatant pH reached a designated value (3.7, 4.0, and 4.4). The protonated TOCN was recovered quantitatively as the sediment after centrifugation, and used for catalytic reactions in Figs 2–4. The prepared protonated TOCN suspension (15 mL, 110 mg by dry weight of TOCN) was mixed with dioxane (15 mL; the final concentration of TOCNs was 0.37 w/v%), and then the hydrolysis reaction was started by adding acetal (1.0 mmol). The resultant mixture was continuously stirred at a designated temperature. The conversions of **1**–**7** were monitored by analyzing aliquot samples, using a supercritical fluid chromatography system (ACQUITY UPC^2^ system; Nihon Waters, Tokyo, Japan) equipped with a Torus 2-PIC column. The conversion was calculated from the peak areas of acetals and aldehydes in the supercritical fluid chromatograms on the basis of calibration curves. The reaction of **8** was monitored by thin layer chromatography. The pH values of the other acid catalysts were adjusted by adding 0.1 M aqueous HCl or 0.1 M NaOH solution, and then the same reaction was performed for comparison. The detailed sample preparation for Table 1 is described in the Supplementary Information.

### Data availability statement

All the data generated and/or analyzed during the current study are included in this article and the Supplementary Information file, and are available from the corresponding author on reasonable request.

## Electronic supplementary material

Supplementary Information

## References

[1] Habibi Y, Lucia LA, Rojas OJ (2010). Cellulose nanocrystals: chemistry, self-assembly, and applications. Chem. Rev..

[2] Holt BL, Stoyanov SD, Pelan E, Paunov VN (2010). Novel anisotropic materials from functionalised colloidal cellulose and cellulose derivatives. J. Mater. Chem..

[3] Klemm D (2011). Nanocelluloses: a new family of nature-based materials. Angew. Chem. Int. Ed..

[4] Giese M, Blusch LK, Khan MK, MacLachlan M (2015). Functional materials from cellulose-derived liquid-crystal templates. J. Angew. Chem. Int. Ed..

[5] Lin N, Huang J, Dufresne A (2012). Preparation, properties and applications of polysaccharide nanocrystals in advanced functional nanomaterials: a review. Nanoscale.

[6] Eyley S, Thielemans W (2014). Surface modification of cellulose nanocrystals. Nanoscale.

[7] Klemm D, Heublein B, Fink HP, Bohn A (2005). Cellulose: fascinating biopolymer and sustainable raw material. Angew. Chem. Int. Ed..

[8] Zhu H (2016). Wood-Derived Materials for Green Electronics, Biological Devices, and Energy Applications. Chem. Rev..

[9] Nechyporchuk O, Belgacem MN, Bras J (2016). Production of cellulose nanofibrils: A review of recent advances. Ind. Crops Prod..

[10] Fukuzumi H (2009). Transparent and high gas barrier films of cellulose nanofibers prepared by TEMPO-mediated oxidation. Biomacromolecules.

[11] Aulin C, Gällstedt M, Lindström T (2010). Oxygen and oil barrier properties of microfibrillated cellulose films and coatings. Cellulose.

[12] Fujisawa S (2011). Preparation and characterization of TEMPO-oxidized cellulose nanofibril films with free carboxyl groups. Carbohydr. Polym..

[13] Fortunati E (2012). Multifunctional bionanocomposite films of poly(lactic acid), cellulose nanocrystals and silver nanoparticles. Carbohydr. Polym..

[14] Eichhorn SJ (2010). Review: current international research into cellulose nanofibres and nanocomposites. J. Mater. Sci..

[15] Wei H, Rodriguez K, Renneckar S, Vikesland PJ (2014). Environmental science and engineering applications of nanocellulose-based nanocomposites. Environ. Sci. Nano.

[16] Olsson RT (2010). Making flexible magnetic aerogels and stiff magnetic nanopaper using cellulose nanofibrils as templates. Nat. Nanotechnol..

[17] Moon RJ (2011). Cellulose nanomaterials review: structure, properties and nanocomposites. Chem. Soc. Rev..

[18] Dong H, Snyder JF, Williams KS, Andzelm JW (2013). Cation-Induced Hydrogels of Cellulose Nanofibrils with Tunable Moduli. Biomacromolecules.

[19] Nemoto J, Saito T, Isogai A (2015). Simple Freeze-Drying Procedure for Producing Nanocellulose Aerogel-Containing, High-Performance Air Filters. ACS Appl. Mater. Interfaces.

[20] Liew SY, Thielemans W, Walsh DA (2010). Electrochemical Capacitance of Nanocomposite Polypyrrole/Cellulose Films. J. Phys. Chem. C.

[21] Hsieh MC, Kim C, Nogi M, Suganuma K (2013). Electrically conductive lines on cellulose nanopaper for flexible electrical devices. Nanoscale.

[22] Astruc D, Lu F, Aranzaes JR (2005). Nanoparticles as Recyclable Catalysts: The Frontier between Homogeneous and Heterogeneous Catalysis. Angew. Chem. Int. Ed..

[23] White RJ (2009). Supported metal nanoparticles on porous materials. Methods and applications. Chem. Soc. Rev..

[24] Polshettiwar V, Varma RS (2010). Green chemistry by nano-catalysis. Green Chem..

[25] Kaushik M, Moores A (2016). Review: nanocelluloses as versatile supports for metal nanoparticles and their applications in catalysis. Green Chem..

[26] Lin X (2011). Platinum nanoparticles using wood nanomaterials: eco-friendly synthesis, shape control and catalytic activity for *p*-nitrophenol reduction. Green Chem..

[27] Zhou P (2012). Bacteria Cellulose Nanofibers Supported Palladium(0) Nanocomposite and Its Catalysis Evaluation in Heck Reaction. Ind. Eng. Chem. Res..

[28] Wu X (2014). Green synthesis and formation mechanism of cellulose nanocrystal-supported gold nanoparticles with enhanced catalytic performance. Environ. Sci. Nano.

[29] Kaushik M (2015). Cellulose Nanocrystals as Chiral Inducers: Enantioselective Catalysis and Transmission Electron Microscopy 3D Characterization. J. Am. Chem. Soc..

[30] Isogai A, Saito T, Fukuzumi H (2011). TEMPO-oxidized cellulose nanofibers. Nanoscale.

[31] Koga H (2010). Topochemical synthesis and catalysis of metal nanoparticles exposed on crystalline cellulose nanofibers. Chem. Commun..

[32] Azetsu A, Koga H, Isogai A, Kitaoka T (2011). Synthesis and Catalytic Features of Hybrid Metal Nanoparticles Supported on Cellulose Nanofibers. Catalysts.

[33] Koga H (2012). Topological loading of Cu(I) catalysts onto crystalline cellulose nanofibrils for the Huisgen click reaction. J. Mater. Chem..

[34] Bendi R, Imae T (2013). Renewable catalyst with Cu nanoparticles embedded into cellulose nano-fiber film. RSC Adv..

[35] Prathap KJ, Wu Q, Olsson RT, Dine P (2017). Catalytic Reductions and Tandem Reactions of Nitro Compounds Using *in Situ* Prepared Nickel Boride Catalyst in Nanocellulose Solution. Org. Lett..

[36] Serizawa T, Sawada T, Wada M (2013). Chirality-specific hydrolysis of amino acid substrates by cellulose nanofibers. Chem. Commun..

[37] Serizawa T, Sawada T, Okura H, Wada M (2013). Hydrolytic Activities of Crystalline Cellulose Nanofibers. Biomacromolecules.

[38] Tsutsumi Y (2014). Nanofibrillar Chitin Aerogels as Renewable Base Catalysts. Biomacromolecules.

[39] Kühbeck D, Saidulu G, Reddy KR, Díaz DD (2012). Critical assessment of the efficiency of chitosan biohydrogel beads as recyclable and heterogeneous organocatalyst for C–C bond formation. Green Chem..

[40] Mahé, O., Brière, J. F. & Dez, I. Chitosan: An Upgraded Polysaccharide Waste for Organocatalysis. *Eur*. *J*. *Org*. *Chem*. 2559–2578 (2015).

[41] Su C, Loh KP (2013). Carbocatalysts: Graphene Oxide and Its Derivatives. Acc. Chem. Res..

[42] Huang YB, Fu Y (2013). Hydrolysis of cellulose to glucose by solid acid catalysts. Green Chem..

[43] Zhang F (2014). Amine-Functionalized GO as an Active and Reusable Acid–Base Bifunctional Catalyst for One-Pot Cascade Reactions. ACS Catal..

[44] Hattori, H. & Ono, Y. Solid Acid Catalysis: From Fundamentals to Applications (CRC Press, 2015).

[45] Mazeau K, Vergelati C (2002). Atomistic Modeling of the Adsorption of Benzophenone onto Cellulosic Surfaces. Langmuir.

[46] Perez DDS (2004). Theoretical and Experimental Studies on the Adsorption of Aromatic Compounds onto Cellulose. Langmuir.

[47] Boraston AB, Bolam DN, Gilbert HJ, Davies GJ (2004). Carbohydrate-binding modules: fine-tuning polysaccharide recognition. Biochem. J..

[48] Mazeau K, Wyszomirski M (2012). Modelling of Congo red adsorption on the hydrophobic surface of cellulose using molecular dynamics. Cellulose.

[49] Payne CM (2015). Fungal Cellulases. Chem. Rev..

[50] Suganuma S (2008). Hydrolysis of Cellulose by Amorphous Carbon Bearing SO_3_H, COOH, and OH Groups. J. Am. Chem. Soc..

[51] Onda A, Ochi T, Yanagisawa K (2008). Selective hydrolysis of cellulose into glucose over solid acid catalysts. Green Chem..

[52] Wu Y (2010). Microwave-assisted hydrolysis of crystalline cellulose catalyzed by biomass char sulfonic acids. Green Chem..

[53] Shuai L, Pan X (2012). Hydrolysis of cellulose by cellulase-mimetic solid catalyst. Energy Environ. Sci..

[54] Fujisawa S, Togawa E, Kuroda K (2017). Facile Route to Transparent, Strong, and Thermally Stable Nanocellulose/Polymer Nanocomposites from an Aqueous Pickering Emulsion. Biomacromolecules.

[55] Gestranius M (2017). Phase behaviour and droplet size of oil-in-water Pickering emulsions stabilised with plant-derived nanocellulosic materials. Colloids and Surfaces A: Physicochem. Eng. Aspects.

[56] Kondo T, Kose R, Naito H, Kasai W (2014). Aqueous counter collision using paired water jets as a novel means of preparing bio-nanofibers. Carbohydr. Polym..

[57] Saito T, Isogai A (2004). TEMPO-Mediated Oxidation of Native Cellulose. The Effect of Oxidation Conditions on Chemical and Crystal Structures of the Water-Insoluble Fractions. Biomacromolecules.

