# Supplementary material for: Interfacial Hydrolysis of Acetals on Protonated TEMPO-oxidized Cellulose Nanofibers
Source: Sci Rep. 2018 Mar 22;8:5021. doi: 10.1038/s41598-018-23381-8 (PMC5864833; doi:10.1038/s41598-018-23381-8)
Supplement: Supplementary file 1 — Supplementary Information [file 41598_2018_23381_MOESM1_ESM.pdf]

Supplementary Information

Interfacial Hydrolysis of Acetals on Protonated TEMPO-oxidized Cellulose Nanofibers

Yuya Tamura, Kyohei Kanomata & Takuya Kitaoka*

Department of Agro-Environmental Sciences, Graduate School of Bioresource and Bioenvironmental Sciences, Kyushu University, 6-10-1 Hakozaki, Higashi-ku, Fukuoka 812-8581, Japan

*e-mail: tkitaoka@agr.kyushu-u.ac.jp

Table of Contents

1. **Materials**
2. **General information on characterization**
3. **Preparation of TOCNs with various carboxy contents**
4. **Dimethoxymethylbenzene hydrolysis at different pH values**
5. **Preparation of mercerized TOCNs**
6. **XRD analysis**
7. **Kinetic analysis of hydrolysis reaction**
8. **TEM images**
9. **Hydrolysis of dimethoxymethylbenzene catalyzed by protonated TOCNs with various carboxy contents**
10. **Substrate synthesis**

1. Materials

Softwood-derived TEMPO-oxidized cellulose nanofibers (TOCNs, 1.1 wt%, COONa = 1.57 mmol g^{-1} of TOCNs) were supplied by Nippon Paper Industries Co., Ltd. (Tokyo, Japan). Carboxylate-free cellulose nanofibers (BiNFi-s, WMa-10002, 2.1 wt%) were purchased from Sugino Machine Limited (Uozu, Japan). The reactions in Figures 2, 3, 4, S2, S5, and S6 were carried out by using TOCNs supplied by Nippon Paper Industries. The reactions in Table 1 were performed using TOCNs prepared in our laboratory by TEMPO-mediated oxidation of physically pulverized CNFs (BiNFi-s, see Preparation of TOCNs in Materials and Methods). Mercerized TOCNs were prepared from TOCNs supplied by Nippon Paper Industries (see Section 5 in the Supplementary Information).

The following chemicals were purchased from Sigma–Aldrich Japan (Tokyo, Japan);

4-bromobenzaldehyde dimethyl acetal	98% purity
(1,1-dimethoxyethyl)benzene	97% purity
4-nitrobenzaldehyde	98% purity
sodium bromide	99% purity
sodium hypochlorite solution (available chlorine 10–15%)	reagent grade
2,2,6,6-tetramethylpiperidine 1-oxyl	98% purity
p-toluenesulfonic acid monohydrate	99% purity

The following chemicals were purchased from Wako Pure Chemical Industries, Ltd. (Osaka, Japan);

acetic acid	guaranteed reagent
benzaldehyde	guaranteed reagent
dimethoxymethylbenzene	guaranteed reagent
N,*N*-dimethylformamide (super dehydrated)	for organic synthesis
dioxane	guaranteed reagent
ethanol	guaranteed reagent
ethyl acetate	guaranteed reagent
hydrochloric acid (35.0–37.0%)	guaranteed reagent
magnesium sulfate, anhydrous	practical grade
methanol	guaranteed reagent
polyacrylic acid (MW = *ca*. 5,000)	Wako 1st grade

potassium carbonate	Wako 1st grade
2-propanol	for HPLC
sodium hydroxide	guaranteed reagent
sodium sulfate	Wako 1st grade
trimethyl orthoformate	Wako 1st grade

The following chemicals were purchased from Tokyo Chemical Industry Co., Ltd. (Tokyo, Japan);

p-anisaldehyde dimethyl acetal	97% purity
carboxymethycellulose sodium salt (n = *ca*. 500)	
diethoxymethylbenezene	98% purity
methyl β-D-glucopyranoside hemihydrate	98% purity
methyl iodide	99.5% purity
4-methyl-2-phenyl-1,3-dioxolane	98% purity
3-phenylpropionaldehyde	93% purity
sodium hydride (60%, dispersion in paraffin liquid)	

All the reagents and solvents were used as received without further purification unless otherwise noted. The accurate concentration of sodium hypochlorite solution was determined by titration. Benzaldehyde was used to make a calibration curve for SFC analysis, which was purified by distillation prior to use. The carboxylate contents of polyacrylic acid and carboxymethycellulose were 11.7 and 2.74 mmol g^{-1}, respectively, which were determined by conductometric titration[1]. Carboxymethycellulose sodium salt was protonated by dissolving in 0.1 M HCl aqueous solution, dialyzed, and freeze-dried prior to use. The water used in this study was purified by an Arium Ultrapure Water System (Sartorius Co., Ltd., Tokyo, Japan).

2. General information on characterization

X-Ray diffraction (XRD) patterns were recorded using an RINT 2000 (Rigaku, Tokyo, Japan) instrument with Ni-filtered Cu Kα radiation (λ = 0.1528 nm) at 40 kV and 20 mA. Scanning was performed at 0.5° min^{-1} with 0.1° intervals. Samples were prepared by pressing freeze-dried TOCNs to make a pellet. The crystallinity index (CrI) was calculated using equation (1), based on the method reported by Segal and co-workers.[2]

$$\text{CrI (\%)} = [(I_{200} - I_{am})/I_{200}] \times 100 \qquad (1)$$

where I_{200} is the intensity at $2\theta = 22.5°$ and I_{am} is that at $2\theta = 18.7°$. The sample crystallite dimensions were calculated using the Scherrer equation:

$$D_{200} = 0.9\lambda/\beta_{1/2}\cos\theta \qquad (2)$$

where D_{200} is the crystallite dimension in the direction normal to the (200) lattice planes, λ is the X-ray radiation wavelength (0.15406 nm), $\beta_{1/2}$ is the full width at half-maximum of the diffraction peak, and θ is the corresponding Bragg angle (11.25°).

Transmission electron microscopy (TEM) was performed using a JEM-2100HCKM microscope (JEOL, Tokyo, Japan), operated at an accelerating voltage of 200 kV, at the Ultramicroscopy Research Center Kyushu University. A 0.05% TOCN dispersion (5 μL) was mounted on a glow-discharged carbon-coated Cu grid. The excess liquid was absorbed with a filter paper, and a negative staining reagent, i.e., 1% sodium phosphotungstate (5 μL), was dropped onto the sample. After standing for 5 min, the excess liquid was absorbed with a filter paper. Deionized water (5 μL) was added dropwise, and removed with a filter paper after 3 min. After air-drying, the TOCNs on the sample grid were observed using the TEM apparatus in bright-field mode.

3. Preparation of TOCNs with various carboxy contents

A series of TOCNs with different carboxy contents were prepared from physically pulverized CNFs (BiNFi-s WMa-10002, 2.1 wt% of water suspension; Sugino Machines Limited, Uozu, Japan) by varying the amount of the oxidant (NaClO). The preparation conditions and results are summarized in Table S1 and Figure S1.

Table S1. Conditions for TOCN preparation from physically pulverized CNFs

Entry	NaClO / mmol g^{-1}	Reaction time / min	Pass number[a]	Carboxy content / mmol g^{-1}
1	0.75	40	120	0.39
2	2.0	40	30	0.94
3	3.0	40	10	1.21
4	4.0	50	10	1.55
5	5.0	70	10	1.81

[a]Number of cycles applied in aqueous counter collision process with high-speed water-jet machine at 245 MPa (Star Burst Labo, Sugino Machines Limited, Uozu, Japan).

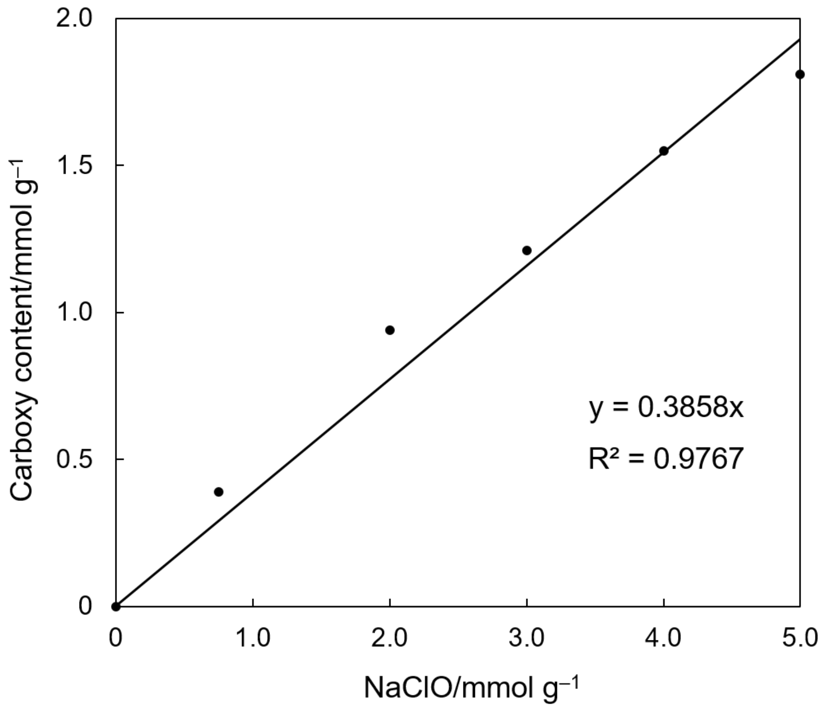

Figure S1. Relationship between amount of NaClO and carboxy content of TOCNs.

4. Dimethoxymethylbenzene hydrolysis at different pH values

(a) Hydrolysis of dimethoxymethylbenzene at the same catalyst loading (adjusted pH 3.7 or 4.4)

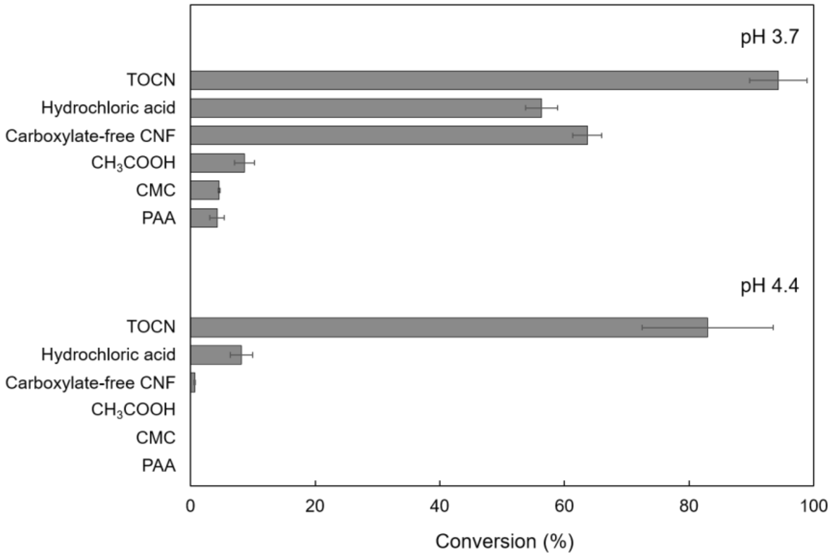

(b) Hydrolysis of dimethoxymethylbenzene at the same catalyst loading (different pH in each)

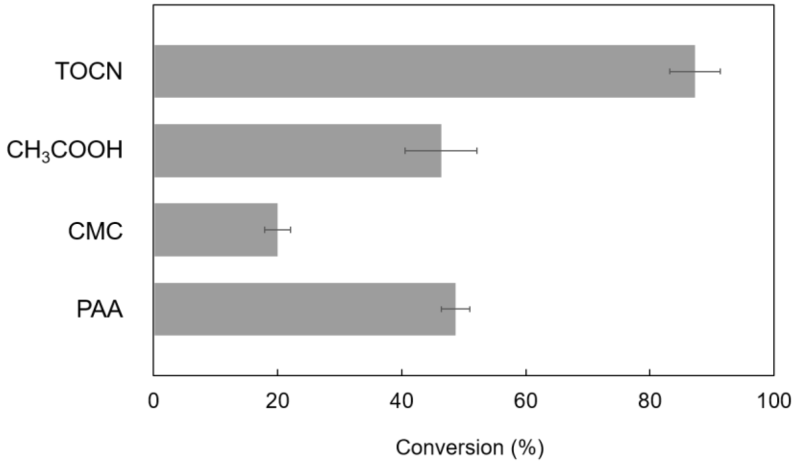

Figure S2. Hydrolysis of dimethoxymethylbenzene using various acid catalysts.

In Figure S2a, the conversion was evaluated at the identical pH value for each catalyst. Each sample having 0.17 mmol of carboxylic acid, which was identical to molar amount of carboxy group in TOCN, was added to water, and then the pH was adjusted to the designated value by adding 0.1 M aqueous HCl or 0.1 M NaOH solution, followed by the addition of dioxane. The dimethoxymethylbenzene hydrolysis reactions were performed with TOCNs or other acid catalysts at room temperature for 2 h at pH 3.7, 4.0, and 4.4 (the data for pH 4.0 are shown in Figure 1). The superiority of the TOCNs is the most obvious at pH 4.4. The TOCNs are superior to other acid catalysts even at pH 3.7, at which the background reaction mediated by HCl is not negligible.

In Figure S2b, the conversion was compared with the same molar catalyst loading but at different pH values for each catalyst. Each sample having 0.17 mmol of carboxylic acid was added to an aqueous solution of HCl at pH 4.0. The consequent pH value was different in each: 4.0 for TOCNs; 3.2 for acetic acid; 3.2 for CMC; and 3.3 for PAA. Dioxane was then added to the solutions, and the resultant mixtures were used for the hydrolysis reaction. CMC considerably aggregated at pH 3.3 in water/dioxane.

5. Preparation of mercerized TOCNs

A TOCN water dispersion (1.1 wt% of water suspension, 2.0 g of TOCNs by dry weight, the Nippon Paper Industries Co. Ltd., Tokyo, Japan) was mixed with 33% aqueous NaOH solution (200 mL), and the resultant mixture was stirred at room temperature for 2 h (final NaOH concentration: 18.8 w/v%), affording a yellowish suspension (final TOCN concentration: 0.5 wt%). The mixture was acidified with 6 M aqueous HCl solution to pH 1.0, and the precipitate was washed repeatedly with water by centrifugation. The precipitate was re-suspended in water (100 mL g^{-1} of cellulose) and neutralized with 2 M aqueous NaOH solution until the pH was 10. The resultant suspension was treated with an ultrasonic homogenizer at 19.5 kHz for 20 min (US-300E; Nihonseiki, Tokyo, Japan) to afford a translucent dispersion (1.3 wt% of mercerized TOCNs). The overall yield was ca. 60%, based on the dry weight of cellulose. The XRD pattern of the prepared mercerized TOCNs clearly shows the cellulose II crystalline structure (Figure S3a); the TEM image shows a nano-ordered rod-like shape (Figure S3b). The carboxy content of the mercerized TOCNs was 1.12 mmol g^{-1}, which was determined by conductometric titration[1].

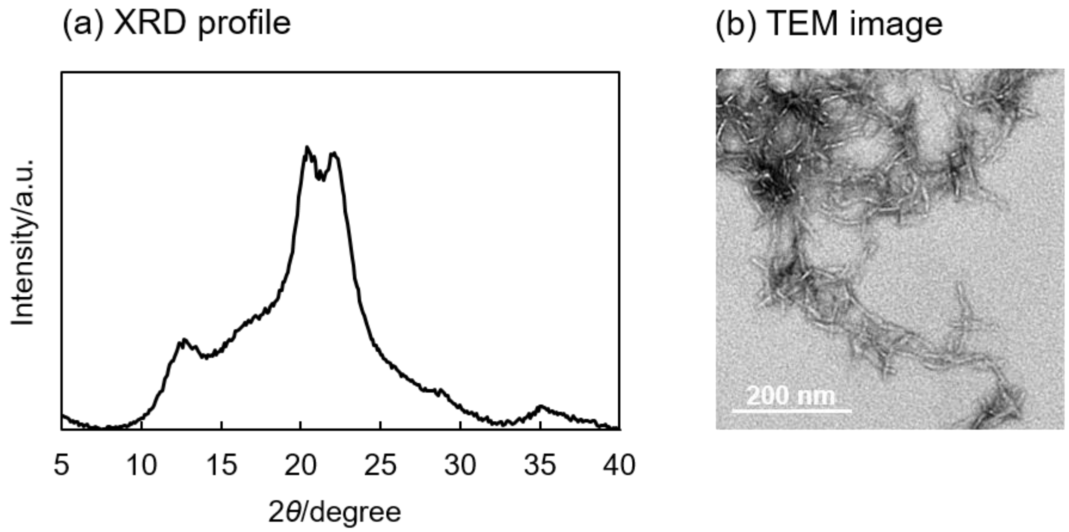

Figure S3. XRD pattern (a) and TEM image (b) of mercerized TOCNs.

6. XRD analysis

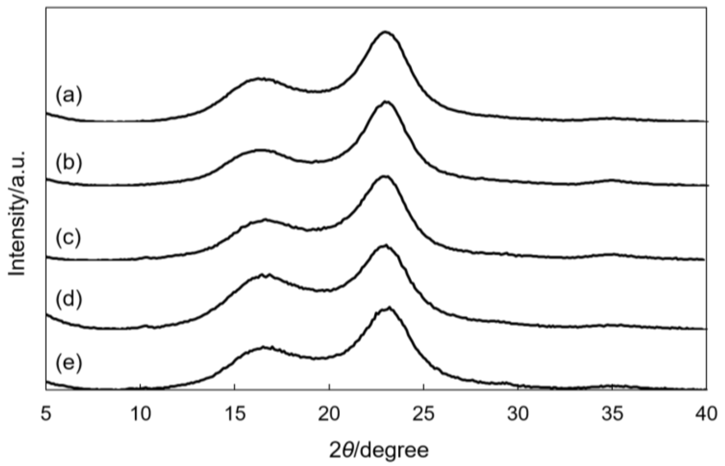

Entry	CrI / %	D_{200} / nm
a	66.5	2.3
b	72.1	2.6
c	64.5	2.3
d	54.4	2.0
e	62.5	2.3

Figure S4. XRD patterns of protonated TOCNs: (a) TOCNs supplied by the Nippon Paper Industries Co., Ltd.; (b) TOCNs recovered after hydrolysis reaction (Table 1, entry 1); (c) TOCNs with carboxy content of 0.39 mmol g^{-1}, prepared from BiNFi-s (Sugino Machines Limited); (d) TOCNs with carboxy content of 1.55 mmol g^{-1}, prepared from BiNFi-s; (e) TOCNs with carboxy content of 1.81 mmol g^{-1}, prepared from BiNFi-s. Preparation of the TOCNs is described in Section 2 above.

7. Kinetic analysis of hydrolysis reaction

The activation energy (E_a) and the frequency factor (A) of the reaction were calculated using the Arrhenius equation:

$$\ln k = \ln A - E_a/RT \qquad (3)$$

where k is the rate constant of the reaction, A is the frequency factor, E_a is the activation energy, R is the gas constant, and T is the temperature.

Table S2. Reaction rate constants of dimethoxymethylbenzene hydrolysis reactions

Temperature / °C	Rate constant k / min^{-1}	
	TOCNs	HCl
4	0.000627	0.000309
10	0.00203	0.000779
20	0.00660	0.00267
40	0.0648	0.0215
60	0.473	0.158

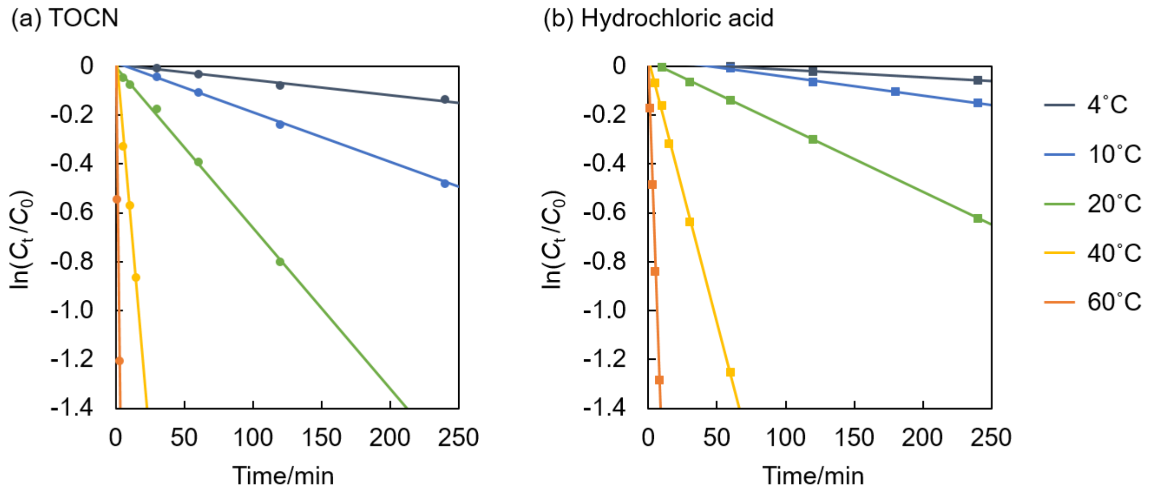

Figure S5. Plots of $\ln(C_t/C_0)$ versus reaction time for dimethoxymethylbenzene hydrolysis reactions at pH 4.0 with (a) TOCNs and (b) HCl.

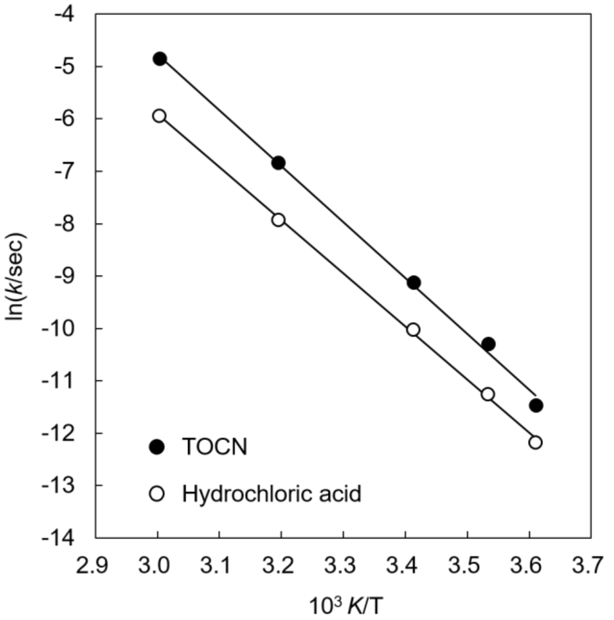

Figure S6. Arrhenius plots for dimethoxymethylbenzene hydrolysis reactions at pH 4.0 with TOCNs and HCl.

Table S3. Kinetic analysis of hydrolysis reactions with TOCNs and HCl

	Activation energy E_a / kJ mol^{-1}	Frequency factor A / s^{-1}
TOCNs	88.8	7.04×10^{11}
HCl	84.2	0.39×10^{11}

8. TEM images

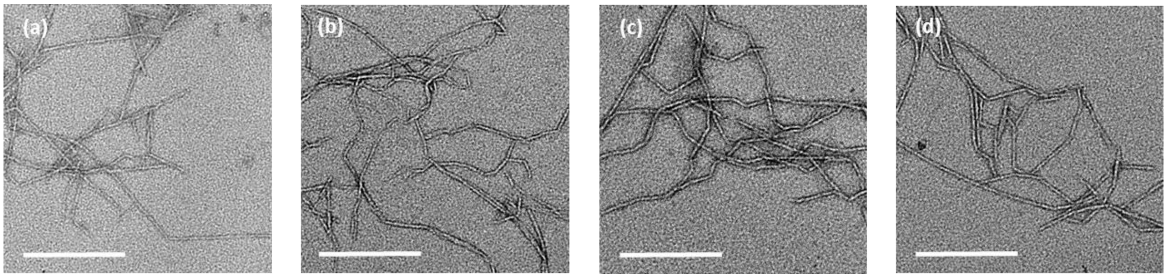

Figure S7. TEM images of TOCNs: (a) TOCNs supplied by the Nippon Paper Industries Co., Ltd.; (b) TOCNs with carboxy content of 0.39 mmol g^{-1}, prepared from BiNFi-s; (c) TOCNs with carboxy content of 1.55 mmol g^{-1}, prepared from BiNFi-s; (d) TOCNs with carboxy content of 1.81 mmol g^{-1}, prepared from BiNFi-s. Scale bars: 200 nm.

9. Hydrolysis of dimethoxymethylbenzene catalyzed by protonated TOCNs with various carboxy contents

For the sample preparation in Table 1, each water suspension of TOCNs with various carboxy contents (0.4–0.7 wt%; 0.39–1.81 mmol g^{-1} in sodium carboxylate form) was separately weighed in a 50-mL centrifugal tube (110, 140, 181, and 436 mg by dry weight of TOCNs), and mixed with 0.1 M HCl solution (20 mL). The mixture was shaken several times, followed by centrifugation at 4,300 *g* for 10 min. After the supernatant was removed, the TOCN precipitate was washed with water, followed by centrifugation again. This treatment was repeated until the supernatant pH became 4.0. The prepared protonated TOCNs were recovered quantitatively as the sediment after centrifugation. Each protonated TOCN suspension (25 mL; 110, 140, 181, and 436 mg by dry weight) was mixed with dioxane (25 mL; the final concentrations of TOCNs were 0.22, 0.28, 0.36, and 0.87 w/v%, respectively), and then the hydrolysis reaction was started by adding acetal (1.0 mmol). The resultant mixture was continuously stirred at a designated temperature. The conversion was monitored by analyzing aliquot samples, using a supercritical fluid chromatography system (ACQUITY UPC^2 system; Nihon Waters, Tokyo, Japan) equipped with a Torus 2-PIC column. The conversion was calculated from the peak areas of acetals and aldehydes in the supercritical fluid chromatograms on the basis of calibration curves.

10. Substrate synthesis

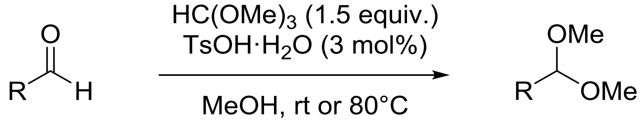

1-(Dimethoxymethyl)-4-nitrobenzene and (3,3-dimethoxypropyl)benzene were synthesized according to the literature method.[3] In brief, TsOH·H_2O (85.6 mg, 0.45 mmol, 3 mol%) was added to a solution of 4-nitrobenzealdehyde or 3-phenylpropionanldehyde (15.0 mmol) and trimethyl orthoformate (2.46 mL, 22.5 mmol, 1.5 equiv) in methanol (20 mL). The mixture was stirred at room temperature or 80°C until completion. The reaction was quenched with triethylamine, and the product was extracted three times with EtOAc. The combined organic layers were washed with brine, dried over Na_2SO_4, and the solvent was removed by evaporation under reduced pressure. The residue was purified by automated flash chromatography (Smart Flash; Yamazen, Osaka, Japan) to give the product. ^{1}H and ^{13}C NMR spectra were recorded with a JNM-ECZ400 instrument (JEOL, Tokyo, Japan) at the Center of Advanced Instrumental Analysis, Kyushu University. For the ^{1}H NMR spectra, the chemical shifts are reported in parts per million from the resonance of tetramethylsilane, which was used as the internal standard (TMS: 0.00 ppm). ^{13}C NMR spectra were recorded with complete proton decoupling, and chemical shifts are reported in parts per million from the solvent resonance as the internal standard ($CDCl_3$: 77.0 ppm). The spectral data for the products were in agreement with those reported in the literature.[4]

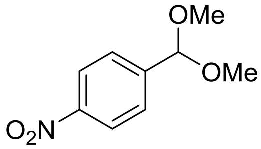

1-(Dimethoxymethyl)-4-nitrobenzene: colorless solid; 92% yield; ^{1}H NMR (400 MHz, $CDCl_3$): δ 3.34 (6H, s), 5.48 (1H, s), 7.64 (2H, d, J = 8.0 Hz), 8.23 (2H, d, J = 8.0 Hz); ^{13}C NMR (100.5 MHz, $CDCl_3$): δ 52.6, 101.4, 123.3, 127.7, 145.0, 147.8.

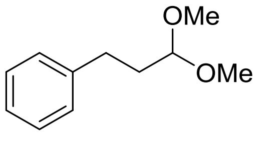

(3,3-Dimethoxypropyl)benzene: pale yellow oil; 59% yield; ^{1}H NMR (400 MHz, $CDCl_3$): δ 1.89–1.95 (2H, m), 2.67 (2H, t, J = 8.0 Hz), 4.37 (2H, t, J = 5.6 Hz), 7.20 (2H, d, J = 7.2

Hz), 7.27–7.30 (3H, m); ^{13}C NMR (100.5 MHz, $CDCl_3$): δ 30.8, 34.0, 52.6, 103.6, 125.8, 128.3 (2C), 141.5.

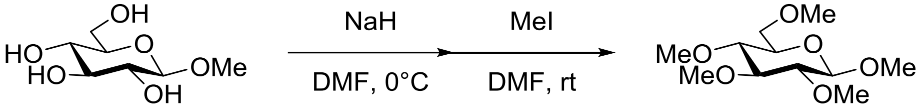

S1

1,2,3,4,6-Penta-*O*-methyl-β-D-glucopyranoside was synthesized from 1-*O*-methyl-β-D-glucopyranoside hemihydrate (**S1**), according to the literature method.[5] In brief, **S1** (3.04 g, 15.0 mmol) and $MgSO_4$ (3.0 g) were added to a 100-mL two-neck round-bottom flask, and the atmosphere was replaced with nitrogen. DMF (10 mL) was added to the flask, and the mixture was stirred at room temperature for 30 min. To another 200-mL two-neck round-bottom flask, NaH (3.6 g, 90 mmol, 60% dispersion) and DMF (50 mL) were added, the atmosphere was replaced with nitrogen, and then the flask was cooled down to 0°C. The DMF solution of **S1** was transferred by using cannula to this flask, and the resultant mixture was stirred for 30 min. Methyl iodide (4.7 mL, 75 mmol) was added in one portion, and the mixture was stirred vigorously at room temperature for 18 h. The reaction was quenched by adding MeOH (5 mL), K_2CO_3 (1.0 g), and water (50 mL). The mixture was extracted three times with EtOAc. The combined organic layers were dried over Na_2SO_4, and the solvent was removed by evaporation under reduced pressure. The residue was purified by automated flash chromatography (Smart Flash; Yamazen, Osaka, Japan) to give the product. The spectral data were in agreement with those reported in the literature.[5]

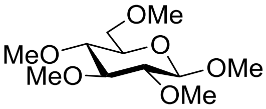

1,2,3,4,6-Penta-*O*-methyl-β-D-glucopyranoside: pale yellow oil; 94% yield; ^{1}H NMR (400 MHz, $CDCl_3$): δ 2.96–3.00 (1H, m), 3.11–3.18 (2H, m), 3.22–3.29 (1H, m), 3.40 (3H, s), 3.52–3.58 (10H, m), 3.62–3.65 (4H, m), 4.14 (1H, d, J = 8.0 Hz); ^{13}C NMR (100.5 MHz, $CDCl_3$): δ 56.9, 59.3, 60.3, 60.4, 60.7, 71.2, 74.5, 79.3, 83.5, 86.4, 104.2.

References

1. Saito, T. & Isogai, A. TEMPO-Mediated Oxidation of Native Cellulose. The Effect of Oxidation Conditions on Chemical and Crystal Structures of the Water-Insoluble Fractions. *Biomacromolecules* **5**, 1983–1989 (2004).
2. Segal, L., Creely, J. J., Martin, A. E. & Conrad, C. M. An Empirical Method for Estimating the Degree of Crystallinity of Native Cellulose Using the X-Ray Diffractometer. *Text. Res. J.* **29**, 786–794 (1956).
3. Pati, K., Gomes, G. D. P., Harris, T. & Alabugin, I. V. Fused Catechol Ethers from Gold(I)-Catalyzed Intramolecular Reaction of Propargyl Ethers with Acetals. *Org. Lett.* **18**, 928–931 (2016).
4. Ito, S., Hayashi, A., Komai, H., Yamaguchi, H., Kubota, Y. & Asami, M. Mesoporous aluminosilicate-catalyzed allylation of carbonyl compounds and acetals. *Tetrahedron* **67**, 2081–2089 (2011).
5. Wever, W. J., Cinelli, M. A. & Bowers, A. A. Visible Light Mediated Activation and *O*-Glycosylation of Thioglycosides. *Org. Lett.* **15**, 30–33 (2013).
